# Provider assessment of the temporomandibular joint in Juvenile idiopathic arthritis: a retrospective analysis from the CARRA database

**DOI:** 10.1186/s12969-024-00968-2

**Published:** 2024-04-08

**Authors:** Anna Costello, Marinka Twilt, Melissa A. Lerman, for the CARRA Registry Investigators

**Affiliations:** 1 Department of Pediatrics, Division of Rheumatology, Children’s Hospital of Philadelphia, 3401 Civic Center Boulevard, Philadelphia, PA 19104 USA; 2 Department of Pediatrics, Division of Rheumatology, Cumming School of Medicine, Alberta Children’s Hospital, University of Calgary, Calgary, AB Canada; 3 Department of Pediatrics, Division of Rheumatology, Children’s Hospital of Philadelphia, University of Pennsylvania, Philadelphia, PA USA

**Keywords:** Juvenile idiopathic arthritis, Temporomandibular joint arthritis, Clinical guidelines, Underdiagnosis

## Abstract

**Background:**

Temporomandibular joint (TMJ) involvement is an often underrecognized complication of juvenile idiopathic arthritis (JIA) that can cause decreased mandibular growth, altered facial morphology, and orofacial pain. It is estimated that the TMJ is affected in 30–45% of children with JIA. Standardized physical examination and imaging evaluations are important in accurately assessing active TMJ arthritis and sequalae. Little is known about the rate at which providers evaluate TMJ involvement in their clinical practice.

**Methods:**

Data were obtained from the Childhood Arthritis and Rheumatology Research Alliance (CARRA) Registry. Data fields related to assessment for TMJ arthritis were added in 2019. Patients were included in the study if they had a diagnosis of JIA and had data recorded between January 2020 and August 2021. Standard descriptive statistics were used to describe demographic and clinical features.

**Results:**

A total of 17,761 visits were reviewed for a total of 7473 patients with JIA. A total of 52.7% of patients had maximal mouth opening (MMO) recorded as finger breadths or total incisal distance (TID). Only 8% had TID measured. A total of 5.0% had MRI with contrast performed. A total of 939 patients had a diagnosis of TMJ arthritis. Of these, 28.5% had an MRI documented, 83% had an MMO documented, and 40% had TID measured. Few patient-level characteristics were statistically related to having MMO assessed. MRI was more likely to be obtained in older and in female patients. MMO was recorded at a given visit > 80% of the time at 17 sites, and it was recorded < 1% of the time at 8 sites. MRIs were infrequently performed at all sites, with 27 sites having no MRIs obtained and only 7 sites having an MRI obtained at > 10% of visits.

**Conclusions:**

MMO is not consistently measured in patients with JIA, and it is rarely measured quantitatively. Similarly, TMJ MRIs are rarely obtained in patients with JIA. Site of care is more associated with TMJ assessments than patient-level characteristics. These data suggest that provider education is needed to improve the assessment of the TMJ in patients with JIA to enable earlier recognition and prevent long-term complications.

## Background

Juvenile idiopathic arthritis (JIA) is the most common rheumatic disease of childhood, affecting about 8 million children worldwide [1]. When JIA causes arthritis of the temporomandibular joint (TMJ), defined as active TMJ arthritis on MRI, it can result in long-term complications, including decreased growth of the mandible, facial asymmetry, retrognathia, and micrognathia [2]. TMJ involvement is associated with long-term orofacial pain, aesthetic problems and poor patient-reported outcomes, including reduced health-related quality of life [3]. Up to 35% of patients with JIA develop dentofacial abnormalities that are significant enough to require orthopedic treatment [4], and others require surgical intervention [5].

Symptoms of TMJ arthritis can include jaw pain, headaches, jaw clicking, and limited mouth opening. Signs of TMJ involvement can include altered mouth opening and facial asymmetry. However, some patients do not show any signs or symptoms even when they have active TMJ arthritis. Patients may develop clinical signs and symptoms only once damage to the joint is present [6]. This makes TMJ arthritis challenging to diagnose in a timely fashion. Given the long-term complications of TMJ arthritis and TMJ involvement, guidelines recommend treating active TMJ arthritis even in the absence of clinical symptoms [7, 8]. Therefore, it is important that clinicians assess the joint as part of standard JIA care even in asymptomatic patients [9].

There are several ways to examine the TMJ, including assessing for pain on palpation of the joint and evaluating frontal facial asymmetry and facial profile convexity. Clinicians should also evaluate mandibular deviation on mouth opening and measure a maximal mouth opening (MMO) [10]. Worsening MMO measurement over time has consistently been shown to correlate with the presence of TMJ arthritis [11]. A standardized technique for examining the TMJ and performing MMO was recently published by a multidisciplinary international task force, the Temporomandibular Joint Juvenile Arthritis Working Group (TMJaw) [10]. Age- and gender-related percentiles for maximal mouth opening have been published in studies of Swiss and Indian healthy children [12, 13]. Maximal mouth opening can be recorded either qualitatively as greater than or less than 3 finger breadths or quantitatively as a total incidence distance (TID) measured in millimeters. While either measurement can demonstrate limited mouth opening suggestive of TMJ arthritis, the total incisal distance can be trended over time and is considered a more objective way to evaluate the joint.

Unfortunately, TMJ arthritis can also be present in patients who maintain a normal mouth opening [14]. As such, imaging remains an important assessment tool. The gold standard imaging to diagnose TMJ arthritis is MRI with contrast, which is recommended by recent guidelines [9]. Contrast is necessary to evaluate features of active TMJ arthritis, including joint effusion, synovial enhancement, and bony edema, in addition to the signs of bony damage that can be seen on noncontrast imaging [15].

While previous studies have reported the prevalence of MMO abnormalities and associated MRI changes in centers in which they are regularly assessed [6], we do not know how frequently these measurment are performed as part of routine rheumatologic patient care. In this study, we use data from the CARRA Registry to determine the frequency at which MMO is evaluated and MRIs are obtained in the real-world care of patients with JIA by pediatric rheumatologists.

## Methods

Data were obtained from the CARRA Registry, which is an observational longitudinal data source for Pediatric Rheumatic Diseases [16]. There are 75 CARRA Registry centers in the US, Canada, and Israel. Disease history and demographics are abstracted at the patient’s enrollment visit. Enrollment visits are often the visit at which they receive their initial diagnosis of arthritis, though patients can also be added to the registry at other points in their disease course. Clinical data are then abstracted every 6 months at routine follow-up visits, and additional unscheduled visits are recorded if a new medication is started. The database has existed since 2015, but data fields related to assessment for TMJ arthritis were added in October 2019. These include: diagnosis of TMJ arthritis (Y/N); date of TMJ arthritis diagnosis; imaging of the TMJ since last visit (Y/N) and, if so, what imaging (MRI with contrast, MRI without contrast, ultrasound, X-ray, CT scan, or other); maximal mouth opening assessment (> 3 finger breadths, < 3 finger breadths, total incisal distance, or not collected); or total incisal distance (in millimeters). These data were collected in the context of routine clinical care, and there is no CARRA Registry protocol for the assessment of the TMJ during clinic visits.

Study population: Subjects were included in the cohort if they had a diagnosis of JIA and had data recorded in the CARRA Registry between January 2020 and August 2021. This included patients from 71 sites of care who participate in the CARRA database. Because some patients transferred care between centers during this period and we were unable to extract which visits occurred at which site of care, visit-level data regarding frequency of MMO and MRI were only performed for patients without any transfers of care.

We evaluated the proportion of patients for whom [1] MMO was recorded and/or [2] MRI was obtained. Total incisal distances (TID) were defined as abnormal if < 30 mm. This cutoff is conservative and represents the lowest end of the published normal ranges for children of any age since we were unable to correlate the measurements to a specific date and, therefore, the patients age at that visit [18]. We assessed outcomes among various subgroups, including by sex, age, JIA category, and race/ethnicity. Age in years was analyzed as an ordinal value (0 to 5, 6 to 12, and 12+). JIA was assessed as a categorical variable, according to ILAR definitions (oligoarticular persistent and extended were not differentiated). In the CARRA database, patients can select multiple options for race and ethnicity. If patients selected multiple options for race, they were included in all selected categories for the analysis. We also performed analysis based on serologic markers, although patients were excluded from this portion of the analysis if the variable was “unknown” or “not completed” in the registry.

## Data analysis

Data were analyzed using R Studio statistical software (version 4.3.2). Descriptive statistics were used. Differences in categorical variables were assessed using the chi^2^ test. Correlations of multiple visit-level characteristics were compared using ANOVA. Statistical significance was defined as a two-tailed p value ≤ 0.05.

This study was reviewed and considered exempt by the Children’s Hospital of Philadelphia Institutional Review Board (IRB 21-018945).

## Results

**Fig. 1:**
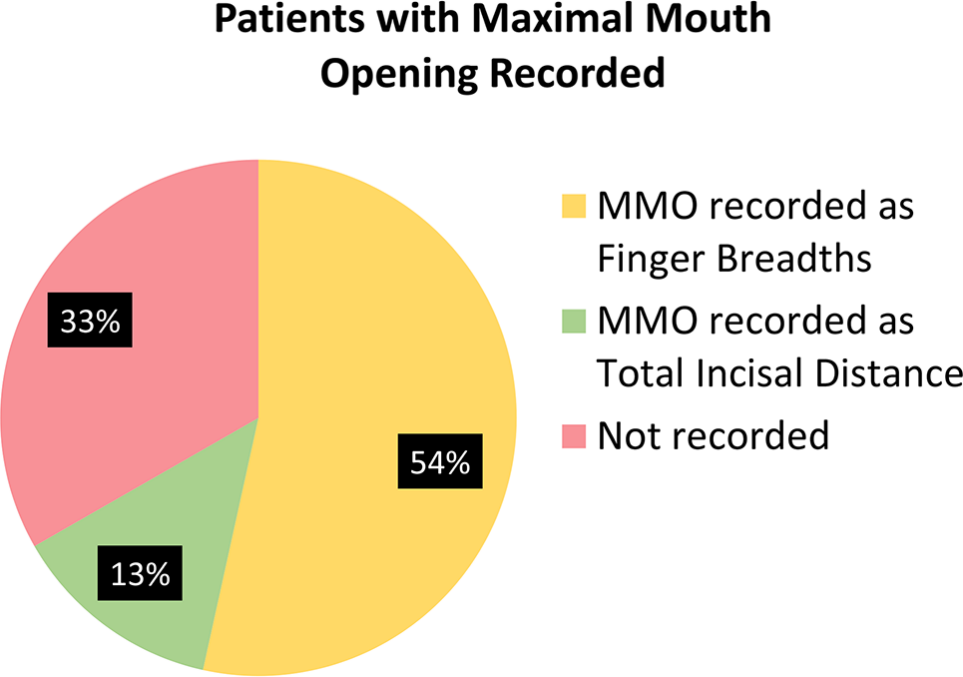
Patients with maximal mouth opening recorded

A total of 7,473 patients with JIA from 59 sites had a total of 17,761 visits in the CARRA registry between January, 2020– August, 2021. Demographic features are described in Table 1. A total of 66.9% of patients had maximal mouth opening (MMO) recorded either as finger breadths or total incisal distance (TID) in millimeters (mm). Of these, only 13% had TIDs recorded. MMO was recorded as less than 3 finger breaths 165 times in 76 patients. This accounted for 0.9% of visits in 1.0% of the patient population (Fig. 1). An abnormal TID was recorded at 45 visits in 37 distinct patients (0.4% of the study population).

**Table 1:** Proportion of patients with maximal mouth opening recorded in the CARRA database

	Total in Sample (n = 7473)	MMO Ever Recorded	p value	MRI with Contrast Ever Recorded	p value
Sex					
Female	5290 (70.8%)	3533 (66.8%)	0.64	297 (5.6%)	< 0.001
JIA Subtype			0.005		< 0.001
ERA*	771 (10.3%)	528 (68.5%)		43 (5.6%)	
Oligoarthritis	2712 (34.3%)	1831 (67.5%)		134 (4.5%)	
Poly (RF neg) *	2227 (29.8%)	1491 (67.0%)		132 (5.9%)	
Poly (RF pos)*	469 (6.2%)	287 (61.2%)		15 (3.2%)	
Psoriatic*	613 (8.2%)	430 (70.1%)		35 (5.7%)	
Systemic*	518 (6.9%)	325 (62.7%)		6 (1.2%)	
Undifferentiated*	156 (2.1%)	109 (69.9%)		7 (4.5%)	
Race and Ethnicity**					
Asian	304	190 (62.5%)		11 (3.6%)	
Black	387	265 (68.5%)		23 (5.9%)	
Hispanic	769	477 (62.0%)		24 (3.1%)	
Middle Eastern	54	28 (51.9%)		2 (3.7%)	
Native American	131	87 (66.4%)		4 (3.1%)	
Native Hawaiian	43	31 (72.1%)		1 (2.3%)	
No answer	166	118 (71.1%)		6 (3.6%)	
Other	101	70 (69.3%)		5 (4.9%)	
White	6036	4082 (67.6%)		310 (5.1%)	
Age			0.765		< 0.001
0 to 5	3317 (44.4%)	2205 (66.5%)		110 (3.3%)	
6–12	2745 (36.7%)	1882 (68.6%)		150 (5.5%)	
12+	1407 (18.8%)	916 (65.1%)		112 (8.0%)	
Diagnosis of TMJ arthritis					
Yes	833 (11.4%)	702 (84.2%)	< 0.001	234 (28.1%)	< 0.001

*Abbreviations* ERA: Enthesitis-related arthritis; Poly: Polyarticular; Psoriatic: Psoriatic arthritis; Systemic: Systemic arthritis; Undifferentiated: Undifferentiated arthritis

**Patients who selected multiple races or ethnicities were included in each category that they selected for a total of 7991 selections

**Fig. 2:**
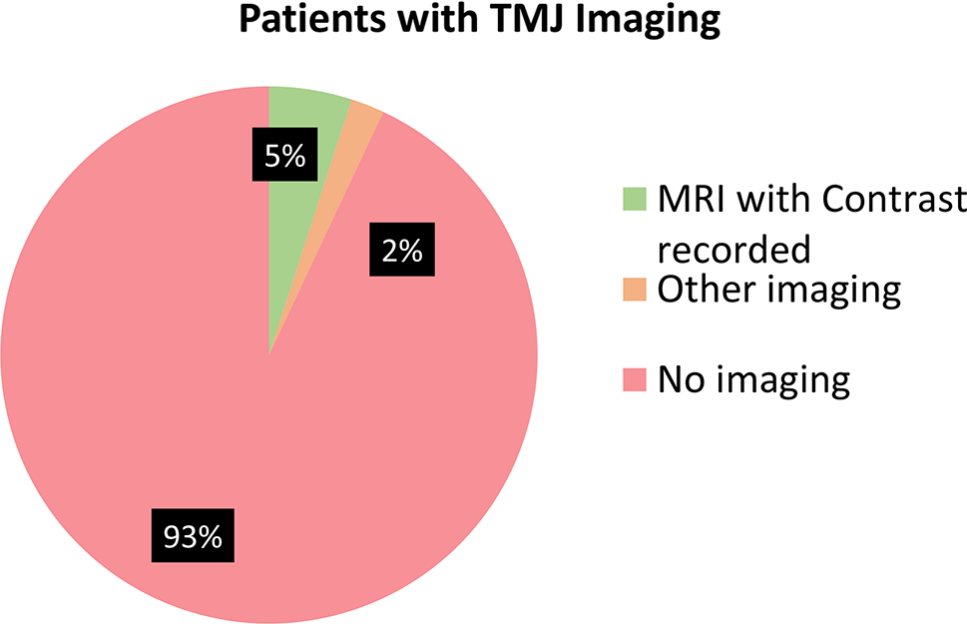
Patients with TMJ imaging

MRI with contrast was obtained a total of 499 times in 5% of the patients in the cohort (Fig. 2). A total of 146 were obtained in patients who did not have a diagnosis of TMJ arthritis documented at the time of that visit. A noncontrast MRI was obtained 197 times.

A total of 1.9% of patients had another type of imaging recorded, including X-rays, cone beam CT scan, or ultrasound. There were 44 visits for 40 patients, during which a non-MRI imaging modality was selected. Of these patients, 7 also had MRIs with contrast obtained, and 23 carried diagnoses of TMJ arthritis.

### Patients with diagnosed TMJ arthritis

A total of 939 patients had a diagnosis of TMJ arthritis (12.6% of the cohort). Of these, 83% had an MMO documented either qualitatively or quantitively; 40% had TID measured, and 28.5% had an MRI documented. During the period of analysis, 51 patients were newly diagnosed with TMJ arthritis. Of these, 84% had MMO recorded, which was evaluated as TID in 51%. 41% had an MRI with contrast.

### Association with patient-level characteristics

MMO was more likely to be assessed in female patients. It was documented in ~ 2/3 of patients in all subtypes without a statistically significant difference between subtypes. There were no statistically significant differences in other demographic characteristics (race and ethnicity or age) (Table 1) or laboratory characteristics (ANA, HLA-B27, RF, or anti-CCP positivity) (Table 2). MRI was more likely to be obtained in female and in older patients. Patients with a diagnosis of TMJ arthritis were significantly more likely to have their MMO recorded and MRI obtained.

**Table 2:** Proportion of patients with TMJ imaging recorded in the CARRA database

	Total with recorded lab in Sample	MMOEver Recorded	p value	MRIwithContrastEver Recorded	p value
ANA Status*			0.53		0.25
Positive	3596 (54.2%)	2435 (67.7%)		199 (5.5%)	
Negative	3039 (45.8%)	2027 (66.7%)		141 (4.6%)	
Anti-CCP**			0.13		0.71
Positive	462 (13.8%)	305 (66.0%)		22 (4.8%)	
Negative	2898 (86.3%)	2004 (69.2%)		164 (5.7%)	
HLA-B27***			0.38		0.50
Positive	638 (17.2%)	458 (71.7%)		33 (5.2%)	
Negative	3063 (82.8%)	2142 (69.9%)		182 (5.9%)	
Rheumatoid Factor****			0.26		0.07
Positive	531 (9.6%)	350 (65.9%)		19 (3.6%)	
Negative	4978 (90.4%)	3405 (68.4%)		277 (5.6%)	

*Abbreviations* ANA: Anti-nuclear antibody. CCP: cyclic citrullinated peptide

*n with recorded ANA 6635, **n with recorded anti-CCP 3360, ***n with recorded HLA-B27 3701, **** n with recorded RF 5509

### Association with visit-level characteristics

**Fig. 3:**
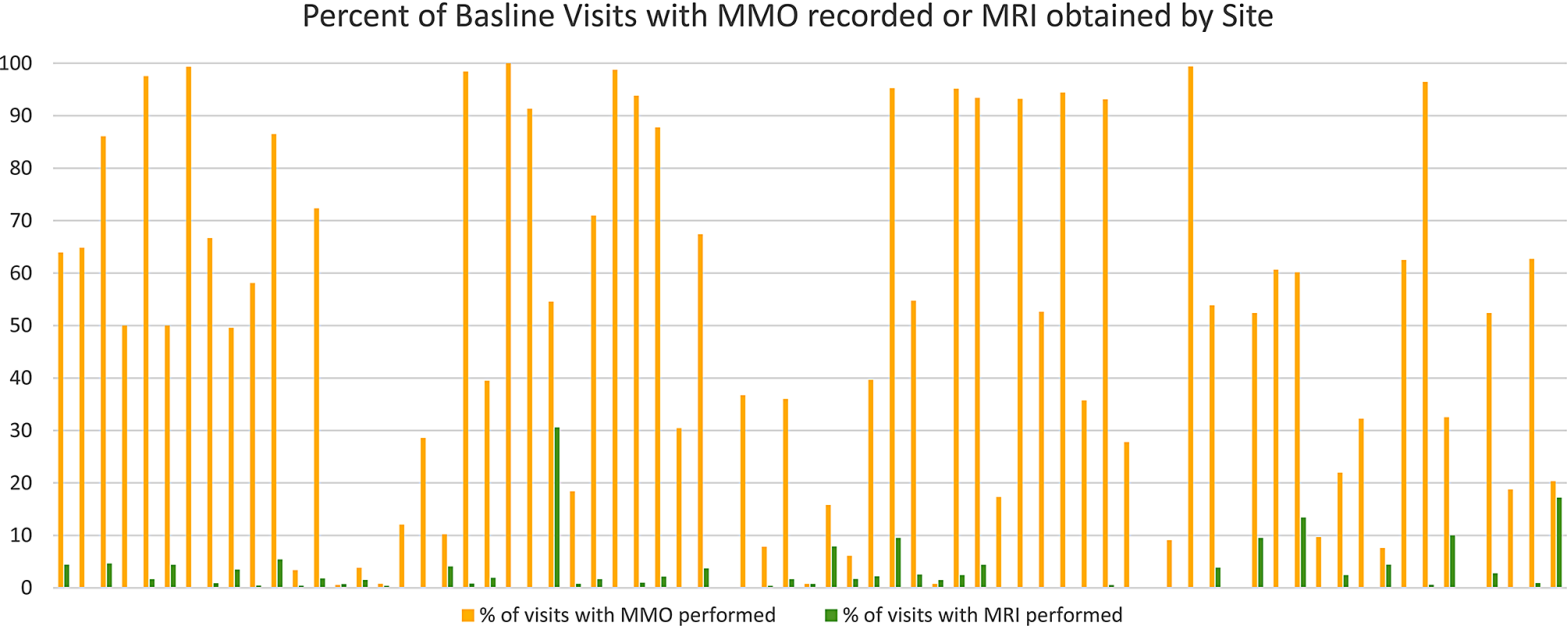
Percent of enrollment visit with MMO recorded of MRI obtained. *Note*: Sites were de-identified in this dataset

It was significantly more likely that MMO was recorded at the patient’s enrollment visit than at follow-up visits. There was significant variance in assessments between the 59 sites. Patients who transferred sites of care were excluded from this analysis to ensure accuracy of site of care. There were 16,644 visits for patients who did not transfer care. MMO was recorded at a given visit on > 80% of the time at 17 sites, and it was recorded < 1% of the time at 8 sites. MRIs were infrequently performed at all sites, with 27 sites having no MRIs obtained and only 7 sites having an MRI obtained at > 10% of visits (Fig. 3). There was no correlation between the sites at which MMO was performed frequently and sites at which MRI was obtained, with no sites being high performers in both categories.

## Discussion

Our data suggest that compared to historical controls, TMJ arthritis is underdiagnosed in CARRA registry patients most likely because there is inadequate assessment of the TMJ via physical examination or imaging. Early diagnosis of TMJ arthritis is important because active arthritis warrants escalation of pharmacologic treatment to limit long-term damage to the joint. It is also important that TMJ involvement (damage and sequalae due to previous TMJ arthritis) is identified, so it can be treated with orthopedic devices or surgery [8].

The true prevalence of active TMJ arthritis and TMJ involvement is likely underdiagnosed in the clinical environment because of the challenges of assessing the TMJ on exam and the need for advanced imaging to fully evaluate for disease activity [17]. The MMO is the only clinical examination finding shown to correlate with active TMJ arthritis in several studies [6, 11]. Patients with JIA should have TID trended over time so that TMJ arthritis can be identified at an early stage. While clinical exam can help identify TMJ arthritis, it likely underestimates the burden of disease. Imaging with MRI is also warranted in patients with JIA [6, 8]. The combination of imaging and repeat clinical exam is the best clinical practice, as the trend of total incisal distances can suggest a change that may indicate the need to obtain or repeat imaging [8].

In our analysis, TMJ arthritis was diagnosed in 12.6% of the cohort, which suggests underdiagnosis of TMJ arthritis based on the range of 30–45% prevalence suggested by prior studies [6, 19]. We suspect that this underdiagnosis is due, at least in part, to limited assessment of the joint in routine clinical practice. Only 67% of patients with JIA had documentation of any form of MMO assessment, and of these, only 13% of patients ever had a TID recorded. Only 5% of patients had an MRI with contrast recorded.

Very few patient-level characteristics were statistically related to having MMO assessed. MMO was more likely to be assessed in female patients, which may be due to female sex being a reported risk factor for TMJ arthritis. There is also strong evidence that young age at JIA onset is a risk factor for TMJ arthritis and damage. More severe and destructive TMJ changes can be seen if TMJ arthritis occurs in young children, as growth can be impaired due to the superficial position of the condylar growth centers [8]. Despite this known risk factor, MMO assessment did not significantly vary with patient age in our analysis.This may be been becuase clinician do not appreciate this important risk or because they have limited skills in MMO assessment in young children (e.g., assessment in the context of missing teeth). Despite evidence suggesting that TMJ arthritis is quite rare in certain subtypes of JIA, such as ERA, and more common in others, such as polyarticular JIA [20], the rate of MMO assessment did not vary between subtypes. This could indicate that providers are not aware of which patients might be at higher risk for TMJ arthritis or that some providers perform this screening routinely while others rarely perform it. While there is some evidence that nonwhite race may be correlated with worse outcomes in patients with JIA [21], there is very little in the literature about any relationship between race and ethnicity and the prevalence of TMJ arthritis. In our analysis, there was no statistically significant difference in the rate of MMO being measured or MRI being obtained between racial and ethnic groups.

As expected, MMO was more commonly documented in patients with a diagnosis of TMJ arthritis. This may have a twofold explanation: providers are more likely to document an MMO in patients with known TMJ disease, and patients with MMOs documented may be more likely to be diagnosed with TMJ arthritis.

Although MMO was consistently performed at some centers, it was typically recorded as greater than or less than 3 finger breadths. The three-finger rule is rarely sensitive enough to appreciate a decrease in MMO indicative of new onset or flare of TMJ arthritis. In this analysis, even when TIDs were collected, there was a wider range of values (3-75 mm) than would be expected, which may be partially attributable to clinicians submitting values that were measured in centimeters that were then transcribed into the registry in millimeters. Alternatively, some clinicians may lack knowledge of how to appropriately measure a TID. Multidisciplinary standardized guidelines for TMJ assessment have been recently published [10], and we hope that the implementation of these guidelines in clinical practice may introduce more reliable measurements in the future.

MMO and TID alone are insufficient to diagnose TMJ arthritis, and MRI remains the gold standard for diagnosis. MRI was rarely recorded in patients in this cohort. There was no significant association with either race/ethnicity or JIA subtype, MRI was more likely to be obtained in female patients and in older patients, possibly because of the recognition that female patients have a higher rate of TMJ arthritis [17]. It is possible that older patients have MRIs obtained more regularly because they do not require sedation. However, since young age is associated with increased risk of TMJ disease [8], it is of concern that imaging is not performed in these children.

MRI with contrast was obtained a total of 499 times in our sample, and 146 of these MRIs were obtained in patients without a documented diagnosis of TMJ arthritis. This suggests that MRIs in some cases were being used diagnostically to determine if TMJ arthritis was present. However, even in those patients with a new diagnosis of TMJ arthritis during the period of our analysis, only 41% had an MRI with contrast of the jaw obtained. It is unclear how providers are diagnosing new active arthritis without contrast enhancement on MRI imaging, the current gold standard to define the disease.

In our analysis, there were 40 patients for whom only non-MRI imaging was performed, including ultrasound, X-ray, CT scan, or other imaging. Seven of these patients also had an MRI with contrast obtained, and 23 of these patients had a diagnosis of TMJ arthritis, so it is possible that this imaging was being used for follow-up of known disease, surgical planning, or other purposes as opposed to diagnosis.

There was tremendous variance in the clinical examination of the TMJ with MMO and MRI between sites of care. This demonstrates that certain providers evaluate the TMJ as part of their routine workflow, while other providers do not. Similarly, there was significant variation in the frequency of obtaining MRIs between sites of care, which also suggests high interprovider variability. Interestingly, there was very little overlap in the sites at which MMO was performed regularly and in the sites at which MRI was obtained regularly. This is challenging to interpret, as we would expect that abnormalities in MMO would trigger MRI assessment, and this likely implies some inaccuracies in data abstraction or documentation.

Overall this data demonstrates that the TMJ is being inadequately assessed in clinical practice. We suspect that this is multifactorial. One barrier may be lack of provider education on this important topic. In fact, recent work has demonstrated that pediatric rheumatologists self-report a lack of confidence in their ability to assess the TMJ and that trainees have limited understanding of how to appropriately measure an MMO [22]. It is also possible that they are not ordering MRIs for their patients due to cost and the need for sedation. Providers may also have concerns about the accuracy of TMJ MRI interpretations and how to act on evidence of TMJ arthritis. Research is needed in these areas to improve clinician confidence in assessing and treating this important joint.

There are several limitations of this study. We relied on observational data from the CARRA Registry, which is a convenience sample. Not all patients who are cared for at an individual site are enrolled in the CARRA Registry, and although we do not have reason to believe that the patients enrolled in the Registry are significantly different from the overall clinic population, there may be some sampling bias. This multicenter cohort mostly captures data from academic centers and from children cared for by pediatric rheumatologists. We would suspect that this may overestimate the degree to which MMO/MRI are being performed, as patients cared for at nonacademic centers may have less access to sedation services for MRIs, and adult rheumatologists may have less awareness of TMJ arthritis. Abstractions of TMJ-related data fields into the database may be incomplete and/or not reflect clinical assessment. The data fields related to the TMJ were newly added at the time of our analysis, and completion rates may increase over time. In most centers, research coordinators collect the data for the CARRA registry, and it might be that providers perform MMO assessment but do not record the information adequately for data extraction. In this case, the likelihood of the provider using the MMO as a longitudinal measurement is minimal. Similarly, it is possible that MRIs are being performed but not extracted into the CARRA Registry. Additionally, while the CARRA Registry collects information on whether the patient has ever had a diagnosis of active TMJ arthritis there is no agreed upon registry definition for active TMJ arthritis.

Our study is the first analysis of provider assessment of the TMJ in a multicenter cohort in real-world clinical practice outside of an orthodontic clinic or a research study. Although the observational data might cause under- or over-estimation, this would not fully account for the low frequency of MMO and MRI assessments that are documented in the registry. Recent studies have found significant TMJ involvement in adult patients with JIA [19, 23], and our data suggest that these sequelae will continue to occur as our current population ages. Our analysis clearly demonstrates that site of care is more associated with TMJ assessments than patient-level characteristics, suggesting that provider practice patterns rather than patient level chracteristics drive these differences. Comprehensive education of providers might improve the assessment of the TMJ in patients with JIA to help prevent long-term complications. This education should include the importance of TID assessment and how to most reliably measure MMO. The value of imaging and the importance of obtaining MRI with contrast in these patients could be another focus for provider education. We hope that further research and educational initiatives can improve accurate and timely diagnosis and treatment for this complicated, underdiagnosed form of arthritis.

## Conclusions

Providers are inconsistently documenting clinical assessment of MMO and TID for TMJ arthritis in patients with JIA and are rarely performing MRIs in these patients. Together, these may contribute to underdiagnosis of TMJ arthritis. Under-recognition of TMJ involvement can lead to long-term sequelae and complications for patients with JIA. Our results highlight the importance of provider education as a step towards improving care of this often neglected joint.

## Data Availability

The data that support the findings of this study are available from Childhood Arthritis and Rheumatology Research Alliance (CARRA) Registry but restrictions apply to the availability of these data, which were used under license for the current study, and so are not publicly available. Data are however available from the authors upon reasonable request and with permission of CARRA.

## Abbreviations

MMO: Maximal mouth opening
JIA: Juvenile idiopathic arthritis
TID: Total Incisal Distance
TMJ: Temporomandibular Joint
CARRA: Childhood Arthritis and Rheumatology Research Alliance

## References

[1] Sacks JJ, Helmick CG, Luo Y, hua, Ilowite NT, Bowyer S (2007). Prevalence of and annual ambulatory health care visits for pediatric arthritis and other rheumatologic conditions in the United States in 2001–2004. Arthritis Rheum.

[2] Glerup M, Stoustrup P, Matzen LH, Rypdal V, Nordal E, Frid P (2020). Longterm outcomes of temporomandibular joints in Juvenile Idiopathic Arthritis: 17 years of followup of a nordic Juvenile Idiopathic Arthritis Cohort. J Rheumatol.

[3] Stoustrup P, Lerman MA, Twilt M (2021). The Temporomandibular Joint in Juvenile Idiopathic Arthritis. Rheum Dis Clin N Am.

[4] Stoustrup P, Glerup M, Bilgrau AE, Küseler A, Verna C, Christensen AE (2020). Cumulative Incidence of Orofacial Manifestations in early juvenile idiopathic arthritis: a Regional, three-year Cohort Study. Arthritis Care Res.

[5] Frid P, Resnick C, Abramowicz S, Stoustrup P, Nørholt SE, Temporomandibular Joint Juvenile Arthritis Work Group TMJaw (2019). Surgical correction of dentofacial deformities in juvenile idiopathic arthritis: a systematic literature review. Int J Oral Maxillofac Surg.

[6] Keller H, Müller LM, Markic G, Schraner T, Kellenberger CJ, Saurenmann RK (2015). Is early TMJ involvement in children with juvenile idiopathic arthritis clinically detectable? Clinical examination of the TMJ in comparison with contrast enhanced MRI in patients with juvenile idiopathic arthritis. Pediatr Rheumatol.

[7] Onel KB, Horton DB, Lovell DJ, Shenoi S, Cuello CA, Angeles-Han ST (2022). 2021 American College of Rheumatology Guideline for the treatment of juvenile idiopathic arthritis: therapeutic approaches for Oligoarthritis, Temporomandibular Joint Arthritis, and systemic juvenile idiopathic arthritis. Arthritis Rheumatol.

[8] Stoustrup P, Resnick CM, Abramowicz S, Pedersen TK, Michelotti A, Küseler A (2023). Management of Orofacial manifestations of Juvenile Idiopathic Arthritis: Interdisciplinary Consensus-based recommendations. Arthritis Rheumatol.

[9] Stoustrup P, Twilt M, Spiegel L, Kristensen KD, Koos B, Pedersen TK (2017). Clinical Orofacial examination in Juvenile Idiopathic Arthritis: International Consensus-based recommendations for monitoring patients in clinical practice and Research studies. J Rheumatol.

[10] Stoustrup P, Herlin T, Spiegel L, Rahimi H, Koos B, Pedersen TK (2020). Standardizing the clinical Orofacial examination in Juvenile Idiopathic Arthritis: an Interdisciplinary, Consensus-based, short screening protocol. J Rheumatol.

[11] Abramowicz S, Susarla HK, Kim S, Kaban LB (2013). Physical findings Associated with active Temporomandibular Joint inflammation in Children with Juvenile Idiopathic Arthritis. J Oral Maxillofac Surg.

[12] Müller L, van Waes H, Langerweger C, Molinari L, Saurenmann RK (2013). Maximal mouth opening capacity: percentiles for healthy children 4–17 years of age. Pediatr Rheumatol Online J.

[13] Patel SM, Patel NH, Khaitan GGA, Thanvi RS, Patel P, Joshi RN (2016). Evaluation of maximal mouth opening for healthy Indian children: Percentiles and impact of age, gender, and height. Natl J Maxillofac Surg.

[14] Stoll ML, Sharpe T, Beukelman T, Good J, Young D, Cron RQ (2012). Risk factors for Temporomandibular Joint Arthritis in Children with Juvenile Idiopathic Arthritis. J Rheumatol.

[15] Larheim TA, Doria AS, Kirkhus E, Parra DA, Kellenberger CJ, Arvidsson LZ (2015). TMJ imaging in JIA patients—An overview. Semin Orthod.

[16] Beukelman T, Kimura Y, Ilowite NT, Mieszkalski K, Natter MD, Burrell G (2017). The new Childhood Arthritis and Rheumatology Research Alliance (CARRA) registry: design, rationale, and characteristics of patients enrolled in the first 12 months. Pediatr Rheumatol Online J.

[17] Koos B, Twilt M, Kyank U, Fischer-Brandies H, Gassling V, Tzaribachev N (2014). Reliability of clinical symptoms in diagnosing Temporomandibular Joint Arthritis in Juvenile Idiopathic Arthritis. J Rheumatol.

[18] Twilt M, Mobers SMLM, Arends LR, ten Cate R, van Suijlekom-Smit L (2004). Temporomandibular involvement in juvenile idiopathic arthritis. J Rheumatol.

[19] Glerup M, Tagkli A, Küseler A, Christensen AE, Verna C, Bilgrau AE (2023). Incidence of Orofacial manifestations of Juvenile Idiopathic Arthritis from diagnosis to Adult Care Transition: a Population-based Cohort Study. Arthritis Rheumatol.

[20] Cannizzaro E, Schroeder S, Müller LM, Kellenberger CJ, Saurenmann RK (2011). Temporomandibular Joint involvement in children with juvenile idiopathic arthritis. J Rheumatol.

[21] Ringold S, Beukelman T, Nigrovic PA, Kimura Y, CARRA Registry Site Principal Investigators (2013). Race, ethnicity, and Disease outcomes in Juvenile Idiopathic Arthritis: a cross-sectional analysis of the Childhood Arthritis and Rheumatology Research Alliance (CARRA) Registry. J Rheumatol.

[22] JIA-Associated TMJ, Arthritis. Idiopathic Condylar Resorption or Anterior Disc Displacement– a Care Provider Survey [Internet]. ACR Meeting Abstracts. [cited 2024 Jan 4]. Available from: https://acrabstracts.org/abstract/jia-associated-tmj-arthritis-idiopathic-condylar-resorption-or-anterior-disc-displacement-a-care-provider-survey/.

[23] De Sonnaville WFC, Speksnijder CM, Zuithoff NPA, Heijstek MW, Wulffraat NM, Steenks MH et al. Clinically established Temporomandibular involvement in adults with juvenile idiopathic arthritis. J Rheumatol. 2023;jrheum.2023– 0204. 10.3899/jrheum.2023-0204 37399466

